# Acid regurgitation associated with persistent cough after pulmonary resection: an observational study

**DOI:** 10.1186/1745-9974-2-9

**Published:** 2006-11-14

**Authors:** Noriyoshi Sawabata, Shin-ichi Takeda, Toshiteru Tokunaga, Masayoshi Inoue, Hajime Maeda

**Affiliations:** 1 Department of Cardiothoracic Surgery, Dokkyo Medical University School of Medicine, Mibu, Tochigi, Japan; 2 Division of Surgery for Chest Diseases, Toneyama National Hospital, Toyonaka, Osaka, Japan; 3 Department of Surgery (E-1), Osaka University, Graduated School of Medicine, Suita, Osaka, Japan

## Abstract

**Background:**

Following a pulmonary resection, some patients suffer from persistent coughing, which may have a relationship with acid regurgitation. Since few physiological studies have been reported regarding this issue, we conducted the present observational study.

**Methods:**

Persistent cough after pulmonary resection (CAP) was defined as non-productive coughing that occurred after a pulmonary resection in patients with stable chest X-ray results and no postnasal drip syndrome, asthma, or history of angiotensin converting enzyme inhibitor administration. A 24-hour esophageal pH monitor was used with patients with coughing (n = 13) and patients with no coughing (n = 4) after undergoing a lobectomy, and the relationship between acid regurgitation and CAP was assessed using symptom association probability.

**Results:**

Based on the results of pH monitoring conducted within 4 weeks of the operation we divided the patients into 3 groups: Type A had frequent gastroesophageal refluxes (>50 occurrences in 24 hours) and frequent coughing (>30 occurrences in 24 hours), Type B had frequent gastroesophageal refluxes and infrequent coughing, and type C had infrequent gastroesophageal refluxes and infrequent coughing. Type A patients (n = 10) were exclusively those with CAP and the symptom association probability was greater than 95%. Five from that group underwent esophageal pH monitoring more than 1 year after surgery and none showed significant improvements in acid regurgitation.

**Conclusion:**

There was a relationship seen between acid regurgitation and CAP in some patients shortly after surgery, while acid regurgitation remained unimproved after improvement of coughing in most of those 1 year after surgery.

## Background

Coughing is a common complication in patients with non-small cell lung cancer after undergoing surgery, as well as phlegm or throat discomfort, wheezing, shortness of breath, and chest pain. Further, it has been reported that approximately 25% of long term survivors (>5 years) suffer from a cough [1], while approximately 50% of those patients suffer from a cough within 1 year of their most recent operation [2]. Extraction of the branches of the vagus from the tracheo-bronchial tract may explain the condition [3-6], though exposed bronchial suture ends [7], lymph node resection [2], hinging of the bronchus, elevation of the diaphragm, unilateral loss of lung volume, and deformity of the residual lung are also possible causes. In addition, acid regurgitation has been proposed [8], as it has been attributed to coughing as well as phlegm or throat discomfort, wheezing, and shortness of breath [9].

Observational and empiric studies of coughing after pulmonary resection (CAP) have been conducted, and it has been proposed that some cases of persistent CAP are caused by acid regurgitation [2]. However, more definitive results regarding the relationship between those conditions are needed. Using esophageal acid monitoring, one of the most sensitive and specific techniques used to diagnose acid regurgitation, we conducted a physiological study of patients with CAP following a lobectomy procedure.

## Methods

### Patients

Seventeen patients who had undergone a lobectomy were selected, of whom 13 had a persistent cough and 4 had no coughing. None of these patients had chronic bronchitis or a diagnosis of gastroesophageal reflux disease (GERD) before surgery, and all symptoms had become manifest after the pulmonary resection procedure. Persistent CAP was defined as non-productive coughing that occurred after the operation in patients with stable chest X-ray results, as well as no postnasal drip syndrome, asthma, or history of angiotensin converting enzyme inhibitor administration, as those are reported to be causes of chronic coughing [10]. Patient characteristics by CAP status are shown in Table 1. At the time of 24-hour pH monitoring, there was no evidence of cancer relapse in any of the patients.

**Table 1:** Characteristics of patients by status of post-operative cough.

Variables	Cough (+)	Cough (-)
Total no.	13	4
Onset		
More than 7 days of OP	13	
Age in years		
Median (range)	66 (48–72)	66(36–68)
Gender		
Male	6	2
Female	7	2
Disease		
Lung cancer	13	4
Surgery		
Lobectomy	13	4
Post-operative days		
Median (range)	18 (15–26)	18(18–24)
Mediastinal lymph node resection		
Yes	13	4
Operation side		
Right	9	2
Left	4	2
Height (cm)		
Median (range)	161 (149–172)	159(158–177)
Weight (kg)		
Median (range)	56 (45–68)	50(45–75)
BSA (cm^2^)		
Median (range)	1.6 (1.4–1.8)	1.5(1.4–1.9)
BMI		
Median (range)	23.0 (18.2–25.9)	23.3(17.8–23.9)

OP: operation

### 24-hour esophageal pH monitoring

All of patients had stopped using proton pomp inhibitors and/or H2-blockers for more than 7 days. A 24-hour esophageal pH study was performed using a Disitrapper 550-1 (Meditoronic LA. USA). A pH electrode was placed 5 cm above the upper border of the lower esophageal sphincter through the nose. Coughing was chosen as the symptom to be recorded and each patient was instructed to push the record button when coughing occurred during the monitoring period. The time trends of esophageal pH (number of reflux occurrences and percentage of time that expectorant was at pH <4.0) and coughing were recorded by the machine. The recorded data were analyzed using computer software (POLYGRAM 98 pH testing system, Medtronic, Skovlunde, Denmark). Monitoring was also performed more than 1 year after the operation in 5 patients in the Type A group, as explained in the Results.

### Symptom analysis

The severity of persistent CAP was analyzed using a visual analog scale (VAS), with a minimum of 0 and maximum of 10 for the number of coughs that occurred during an occurrence of coughing. We also assessed severity by the number of occurrences and duration of expectorant at pH <4. Symptom association probability was calculated using the POLYGRAM 98 software application.

### Symptom association probability

To calculate symptom association probability, we used a contingency table [11], in which the frequency of occurrence of all 4 possible combinations (asymptomatic and symptomatic 2-minute episodes with and without reflux) was recorded. In the symptom analysis of 24-hour esophageal pH data, a time window beginning at 2 minutes before the onset of the symptom incident and ending at its onset provided optimal results [12]. A 2-minute period was considered to be reflux-positive when either a fall in pH greater than 4 units lasted for 5 seconds or more or a fall in pH greater than 1 unit within 5 seconds had occurred. Likewise, all 2-minute periods preceding the onset of symptom episodes were analyzed for the presence of reflux, and then classified as reflux-positive or reflux-negative. Subsequently, a contingency table was constructed that contained 4 fields: the number of symptomatic reflux-positive 2-minute periods (S+R+), the number of asymptomatic reflux-positive 2-minute periods (S-R+), the number of symptomatic 2-minute periods without reflux events (S+R-), and the number of asymptomatic 2-minute periods without reflux events (S-R-). Fisher's exact test was used to calculate the probability (p value) that the observed association between reflux and symptoms occurred by chance [12]. The symptom association probability was calculated using the formula (1.0 - p) × 100%. These calculations were performed by the pH monitoring system.

### Statistical analysis

Measured values are expressed as the mean+/-significant difference. Comparisons between number of refluxes, percent of time at pH <4, VAS, and number of coughing occurrences at less than 4 weeks in all of the patients, and then again more than 1 year after surgery in 5 of the patients, were performed using unpaired t-tests. The results were considered to be significant when the p value was less than 0.05.

## Results

**Figure 1:**
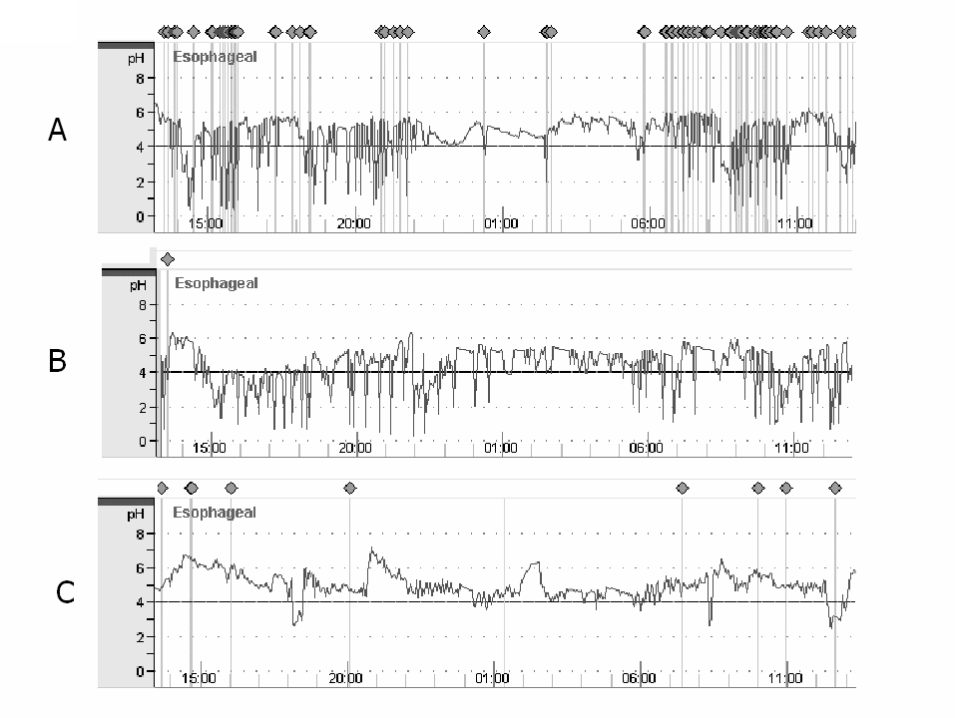
Results of 24-hour esophageal pH monitoring. Based on the results, we divided the patients into 3 groups: type A had frequent acid regurgitation (>50 occurrences) and frequent coughing (>30 occurrences), type B had frequent acid regurgitation (>50 occurrences) and infrequent coughing (<30 occurrences), and type C had infrequent acid regurgitation (<50 occurrences) and infrequent coughing (<30 occurrences).

Detailed information regarding the patients examined is shown in Table 2. None of the patients were obese. A mediastinal lymph node resection was carried out in all patients. Based on the pH monitoring results (Figure 1), we divided the patients into 3 groups: Type A had frequent gastroesophageal refluxes (>50 occurrences in 24 hours) and frequent coughing (>30 occurrences in 24 hours), and were determined to have CAP; Type B had frequent gastroesophageal refluxes and infrequent coughing (without persistent cough after pulmonary resection); and Type C had infrequent gastroesophageal refluxes and infrequent coughing that ceased during monitoring.

**Table 2:** Results of 24-hour pH monitoring in patients with CAP within 4 weeks of the operation.

Case	Age	Sex	Post-OP Cough	Height (cm)	Weight (kg)	BSA (cm^2^)	BMI (kg/m^2^)	OP site	OP	POD	Mediastinal LNRS	%FEV1.0	Smoking
1	48	F	YES	149	51	1.4	23.0	R	L	15	Yes	80.1	Never
2	70	F	YES	152	45	1.4	19.5	R	L	17	Yes	65.3	Never
3	64	F	YES	154	52	1.5	21.9	R	L	17	Yes	96.8	Never
4	69	M	YES	172	64	1.7	21.6	R	L	17	Yes	83.7	Never
5	62	M	YES	164	52	1.6	19.3	R	L	18	Yes	70.2	Never
6	63	F	YES	158	50	1.5	20.0	L	L	18	Yes	50.4	Former
7	66	F	YES	150	58	1.5	25.8	R	L	20	Yes	45.8	Former
8	72	M	YES	165	54	1.6	19.8	R	L	24	Yes	54.0	Current^¶^
9	53	F	YES	162	48	1.5	18.2	R	L	26	Yes	71.4	Never
10	52	F	YES	158	56	1.6	22.4	L	L	21	Yes	89.3	Never
11	66	M	YES	162	68	1.7	25.9	R	L	21	Yes	69.5	Never
12	68	M	YES	161	60	1.6	23.1	L	L	19	Yes	51.4	Former
13	72	M	YES	170	65	1.8	22.5	L	L	21	Yes	40.1	Former
14	66	F	NO^†^	158	50	1.5	20.0	R	L	21	Yes	88.7	Never
15	68	M	NO^†^	177	75	1.9	23.9	R	L	18	Yes	72.4	Former
16	68	M	NO^†^	159	45	1.4	17.8	L	L	24	No	50.8	Current^¶^
17	36	F	NO^†^	159	59	1.6	23.3	L	L	18	No	80.3	Never

^†^No diagnosis of or medication for gastroesophageal reflux disease, ^¶^No symptoms of chronic bronchitis

The results of 24-hour esophageal pH monitoring, coughing occurrence, and VAS for these patients are shown in Table 3. In the Type A group, the symptom association probability was greater than 95% in all 10 cases (100%). In addition, we carried out therapeutic intervention using a proton pomp inhibitor (lansoprazole) and prokinetic agent (mosapride) in all 10 patients in the Type A group, which resulted in improved coughing in 8 cases and stable coughing in 2.

**Table 3:** Results of 24-hour pH monitoring.

Case	No. of reflux occurrences^#^	Time pH <4 (min)^#^	Time pH <4 (%)	Coughing (VAS)	No. of coughing occurrences^#^	Result of pH monitor (group)	SAP
1	57	15	1	6	43	A	97.0
2	86	63	4	10	89	A	100.0
3	126	49	3	6	86	A	99.9
4	263	143	10	10	58	A	100.0
5	195	472	33	3	40	A	97.5
6	443	572	40	4	186	A	99.9
7	110	98	7	6	46	A	97.5
8	193	207	14	3	43	A	100.0
9	310	145	10	5	95	A	99.9
10	227	153	11	8	156	A	99.7
11	131	29	2	5	20	C	80.6
12	43	58	4	3	8	B	60.1
13	38	41	3	3	7	B	61.4
14	285	273	19	0	1	B	69.9
15	256	223	15	0	1	B	69.9
16	413	659	46	0	0	B	0.0
17	441	518	36	1	10	B	81.1

#In 24-hour period, VAS: visual analog scale, SAP: symptom association probability,

Five patients in the Type A group also underwent 24-hour esophageal pH monitoring more than 1 year after the operation. Comparisons between the results obtained within 4 weeks of the operation and those from more than 1 year after surgery are shown in Table 4 and Figure 2. In the latter monitoring results, the number of acid regurgitation occurrences and percent of time at pH less than 4 were not improved significantly, though coughing severity was improved.

**Table 4:** Results of 24-hour esophageal pH monitoring more than 1 year after surgery in patients with CAP originally observed within 4 weeks after the operation.

Case	No. of reflux occurrences^#^	Time pH <4 (min)^#^	Time pH <4 (%)	Coughing (VAS)	No. of coughing occurrences^#^	Result of pH monitor (group)
3	133	53	3.7	0	1	B
5	180	382	28.9	0	0	B
6	422	188	13.1	0	2	B
7	197	150	10.5	0	0	B
9	214	207	14.4	1	18	B

#In 24-hour period, VAS: visual analog scale, SAP: symptom association probability,

**Figure 2:**
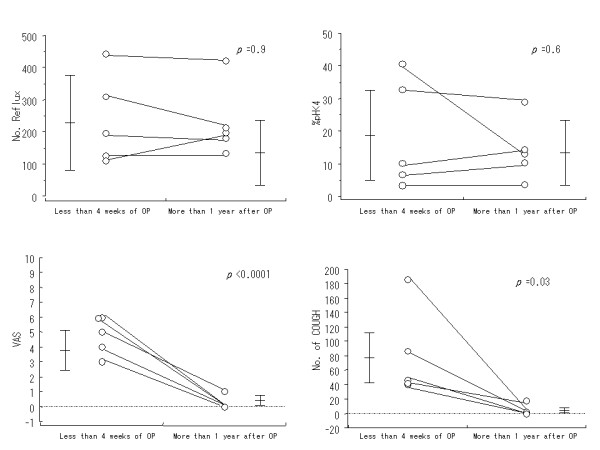
Comparisons of results of 5 patients with coughing within 4 weeks and more than 1 year after undergoing a pulmonary resection procedure. The number of reflux occurrences was 236+/-136 in the patients within 4 weeks after the operation and 229+/-112 at more than 1 year after the operation (p = 0.9). The %pH <4.0 values were 18.7+/-16.8% within 4 weeks after the operation and 14.1+/-9.2% at more than 1 year after the operation (p = 0.6). The visual analog scale results were 4.8+/-1.3 within 4 weeks after the operation and 0.2+/-0.4 at more than 1 year after the operation (p < 0.0001). The numbers of coughing occurrences were 80.0+/-60.2 within 4 weeks after the operation and 4.2+/-7.8 at more than 1 year after the operation (p = 0.03).

## Discussion

There are some negative aspects of patient condition following a pulmonary resection, including loss of lung volume [13], elevation of the diaphragm [14], chest pain [15], and so on. These may lead to a decline in intra-thorax pressure and restriction of diaphragm function. Such conditions explain the acid regurgitation that has been observed to occur soon after surgery in patients who underwent a pulmonary resection.

The major symptoms of gastro-esophageal reflux disease (GERD) are heartburn and acid regurgitation, though some patients have only minor forms of those symptoms [16], which is called silent GERD. The symptoms of silent GERD are wheezing, phlegm or throat discomfort and coughing. Therefore, coughing occurring after a pulmonary resection might be attributed to gastroesophageal reflux.

Coughing after a pulmonary resection has some characteristics, such as delayed onset and non-productive coughing, and occurs in preparing to speech. Observational and empiric investigations in our previous study [2] revealed that the ratio of patients with CAP was 50% within 1 year of the most recent operation and 18% more than 1 year after surgery. Further, gastroesophageal reflux was a significant factor in subchronic patients and 90% of the patients who received empiric therapy saw their coughing symptoms improve after the course of medication. Those results indicated that a secondary change, such as gastroesophageal reflux, caused by surgical intervention is a contributing factor of CAP. However, a more detailed examination of the relationship between CAP and gastroesophageal reflux was considered necessary.

One of the most definitive examinations of acid regurgitation is 24-hour esophageal pH monitoring [10], as it can reveal the relationship between acid regurgitation and coughing incidence, in addition to the numbers of acid regurgitation and coughing occurrences, as well as the incidence of expectorant level at lower than pH 4. In the present study, the number of coughing occurrences was related to the number of acid regurgitation occurrences in patients who showed persistent CAP during monitoring. Therefore, we considered that persistent CAP may be closely related to acid regurgitation, which was supported by our symptom association probability results, as all of the patients who suffered from coughing during monitoring had a symptom association probability value greater than 95%.

These observations can explain the results of our previous observational and empiric study of persistent CAP [2], which revealed that acid regurgitation is a factor in those patients. In that study, we also found that 90% of the patients who received empiric therapy had their coughing symptoms improve after the course of medication. In addition, 8 of 10 patients with persistent coughing after pulmonary resection in the present study saw their coughing improved by administration of a proton pomp inhibitor and prokinetic agent.

The opposing viewpoint must also be considered, i.e. coughing augments acid regurgitation, thus acid regurgitation could be caused by coughing. However, the severity of acid regurgitation in the present patients with a persistent cough after pulmonary resection and frequency of acid regurgitation within 4 weeks of the initial operation did not change when monitored 1 year or more after the operation, regardless of any improvement in coughing severity. In addition, 4 patients with no coughing after the lobectomy procedure also reported acid regurgitation. Thus, there seems to be only a scant contribution by coughing to acid regurgitation.

Improvement of coughing 1 year after surgery is a crucial issue. From our results, it is difficult to conclude that an improvement in acid regurgitation is a contributor to improvement in coughing, as there was little difference in the severity of acid regurgitation seen soon after surgery and more than 1 year later. In addition, degeneration could also be a cause of coughing, such as injury of the vagus, injury of the tracheobronchial area, exposed bronchial suture ends, lymph node resection, hinging of the bronchus, elevation of the diaphragm, unilateral loss of lung volume, and deformity of the residual lung. Therefore, it is important to study the cause of improvement in CAP with multi-focal observations, including acid regurgitation.

This study has some limitations. Owing to irritability caused by trans-nasal insertion of the thin fiber, autonomic nerve conditions may have changed during the examination [4-6]. For example, 2 patients had coughing symptoms improve during the 24-hour esophageal pH monitoring, which resulted in a negative acid regurgitation result. However, the symptom association probability results were very high in the group of patients with a large number of coughing occurrences recorded during monitoring.

## Conclusion

Although there are many possible causes of CAP that should be investigated, a relationship between coughing and acid regurgitation soon after a lobectomy procedure was observed in the present study using a physiological technique with 24-hour esophageal pH monitoring.

## Abbreviations

GERD, gastroesophageal reflux disease

CAP, coughing after pulmonary resection

## Competing interests

The author(s) declare that they have no competing interests.

## Authors' contributions

N.S.; Attending physician, patient observations, esophageal pH monitoring, conducted the study, and wrote the manuscript

S.T.; Attending physician and patient observations

T.T.; Attending physician, patient observations, and esophageal pH monitoring

M.I.; Attending physician and patient observations

H.M.; Attending physician, patient observations, and coordination of the study
